# Leisure and religious activity participation and mental health: gender analysis of older adults in Nepal

**DOI:** 10.1186/1471-2458-7-299

**Published:** 2007-10-22

**Authors:** Ramraj Gautam, Tami Saito, Ichiro Kai

**Affiliations:** 1 Department of Social Gerontology, School of Health Sciences and Nursing, Graduate School of Medicine, University of Tokyo, 7-3-1, Hongo, Bunkyo-Ku, Tokyo 113-0033, Japan

## Abstract

**Background:**

Involvement in activities has been found to be beneficial for improving quality of life and successful aging for older adults. Little is known, however, about the involvement in activities and depression of older adults in Asian developing countries. This study explores whether participation in leisure social and religious activities are related to depression and satisfaction with life in older adults of Nepal. Gender differences are also explored.

**Methods:**

The study sample was derived from a survey which aimed to determine the intergenerational relationships between older adults and their married sons. A cross-sectional quantitative study of older adults sixty years and over in Nepal was conducted with face-to-face interviews using structured instruments. A convenience sample of 489 community dwelling older adults, 247 men and 242 women, were included in the study. The dependent variables, depression and satisfaction with life, were measured by the Geriatric Depression Scale (GDS) and Satisfaction With Life Scale (SWLS) respectively. Age, gender, marital status, education, perceived health, financial satisfaction, social support received and provided by older adults, and social activity were independent variables in the study.

**Results:**

Saying prayers (B = -2.75; p < 0.005), watching television and listening to the radio (B = -1.88; p < 0.05), and participating in physical activity (B = -1.05; p < 0.05) correlated to lower depression for older men, but only watching television and listening to the radio (B = -2.68; p < 0.005) related to lower rates of depression for women. Socializing with others (B = 1.22; p < 0.05) was related to higher satisfaction with life for men, but for women visiting friends (B = 1.29; p < 0.05), socializing with others (B = 1.45; p < 0.005), and watching television and listening to the radio (B = 0.92; p < 0.05) related to improved satisfaction with life. Activity engagement significantly improved mental health in older adults.

**Conclusion:**

Specific activity participation was a significant correlate of lower levels of depression and higher levels of satisfaction with life among older adults in Nepal. The findings explore the need for further research on activity participation in developing countries so that it can be useful for health care practioners and those involved with the activities of aged populations in developing countries.

## Background

Population aging is under- researched and not adopted as a public policy concern in poorer developing countries [1]. The need for additional aging research in South Asia has been identified [2]. Nepal, one of the poorest countries in the world, lies in South Asia, and the population of older adults is increasing faster than the total population over the last three decades [3]. With the growing number of older adults in Nepal, it is important to understand the health issues of the older adult population. Mental health is one aspect of health that needs further exploration in third world countries, such as Nepal [4,5].

Mental health has been clearly established as an important component of public health. Depression is a well recognized mental health problem affecting many older adults that has a negative impact on morbidity, mortality, and quality of life [6,7]. Depression in older adults has been shown to be associated with a loss of physical function [8], poor self-rated health status [9], higher rate of hospitalization [10], suicide [11] and mortality [12]. Both cross-sectional and longitudinal studies have demonstrated an association between psychological factors and symptoms of depression in older adults. Life events, the death of a spouse or loved one, medical illness, functional decline, and lack of social contact were identified in a study as risk factors for depression [13]. Cultural, social, and family relationship factors are also associated with the prevalence of depression and prevent functional decline in older adults [14]. Literature reveals mental well-being in later life is associated with activity participation as well [15]. Researchers have expressed the need of more studies on types of activity involvement among older adults [16].

Activities are important for successful aging. Older adults are involved in a variety of different activities and activities have been found to be beneficial in improving the quality of life and successful aging of older adults [17,18]. Older adults are engaged in social activities more than other age groups, basically because older adults don't have to meet as many obligations as younger people [19]. Social and physical activity participation has been associated with higher life satisfaction, higher self-esteem, lower rates of institutionalization, lower the risk of mortality, and survival [20-22]. Despite depression being a commonly studied mental health problem, very little is known about the activity participation and mental health of older adults in rapidly aging nations of Asia.

There is no well accepted definition of activity in the gerontological literature and different forms of activities are referred to by gerontologists. Activities have been differentially conceptualized and measured in a variety of ways. Various types of activities and their health consequences have been explored in the gerontological literature [23]. The International Classification of Functioning, Disability and Health (ICF) defined social participation as an involvement in life situations, where " involvement may mean being included or engaged in an area of life, being accepted, or having access to needed resources [24]." Activities in this study have been defined as leisure (social and solitary) and religious activities.

Participation in religious activity by older adults has been linked to outcomes such as increased subjective well-being, decreased depression, higher life-satisfaction and self-esteem, better perceived health, and less suicidal ideation and emotional distress [25,26]. A longitudinal study among older Dutch citizens revealed that those older adults who attended church had negative association over time with depressive symptoms [27]. Gender differences in church attendance and its impact on health outcomes are reported in the literature. A study among 4,468 older adults reported frequent church attendance was related to lower prevalence of depression in women but higher prevalence in men [28]. Prayer is identified as a dimension of religiosity appropriate for the life circumstances of older adults [29].

A 10 and 34 year longitudinal study in Sweden showed that though the rate of participation declined over time, previous participation predicted late-life participation [30]. Similarly, another longitudinal study in Canada found that active engagement or participation in social and productive activity predicted outcomes like greater happiness, better functioning, and reduced mortality, whereas solitary activity such as handwork and hobbies predicted happiness, but had no effect on functioning or mortality [18]. Greater participation in solitary activities (working in garden, engaging in hobbies) was significant in reducing the risk of mortality of older adults [31].

The role of marital status in predicting older adults' activity levels is not consistent in the literature. For example, a study found that patterns of social involvement varied by marital status [32]. They suggested that married couples may not need as much social support as widowers because widowers seek more support to compensate for the loss of their spousal intimacy. Another study reported widowed older adults had higher levels of informal social participation than non-widowed, whereas formal social participation levels were comparable [33].

In western societies, research on social activities have focused on engagement in activities after retirement from the labour force. There has been minimal research, however, conducted on how social participation affects the mental health status of older adults in a developing country [34,35]. An ethnographic study in Singapore found that extra-familial social support and opportunities for new experiences in learning and leisure contribute to positive and active living for older adults [34].

The need to look at activity participation in late life among older Asians is recently reported [34]. Although older adults of developing Asian countries are concerned with health issues and financial and caregiving problems, activity participation in late life may have a positive impact on aging-related problems and also enhance life satisfaction [34]. As aging is a new phenomenon in Nepal, no studies have been identified that examine the pattern of involvement of older adults in various activities, and the association of activity involvement with mental health, notably depression and satisfaction with life. In less developed countries like Nepal, where no formal social programs for older adults, such as day care, social clubs, and senior centres are found, participation in activities and involvement by older adults need to be explored.

The present study focuses on activity participation of older adults and its relation to the mental health of older adults in Nepal. The following questions were addressed:

1. Does engaging in leisure (social, solitary) and religious activities correlate with depression and satisfaction with life of older Nepalese adults?

2. Are there differences by gender in activity involvement and depression and satisfaction with life of older Nepalese adults?

## Methods

The study sample was derived from a survey which aimed to examine the relationship between older adults and their married son. The quantitative study was cross-sectional using a face-to-face interviewer-administered structured questionnaire. The data was collected by 10 research assistants during the month of September 2006. The research assistants were trained during a one day intensive seminar regarding interviewing and data collection techniques. The sample was recruited by the research assistants. The inclusion criteria for the sample included those people aged sixty years and above who lived with at least one married son, and was able to communicate well in Nepali. Only one older adult was selected from a household in cases of two or more older adults living in one family, based on their willingness to participate in the research. Interviews were conducted at the respondent's home and other family members were requested to provide privacy during the interview to protect confidentiality of the data. Interviews took place in the absence of other family members.

The sample of older adults was recruited in Kathmandu, Nepal. The 2004 voter list of ward 10 in Kathmandu was used to identify subjects for the sample. Ward 10 was chosen for recruitment because of its centralized position within the city. The voting list recorded 1,539 persons (786 men and 753 women) aged 60 and above of a total population of over 25,000. Nearly 3.9% of the population in Kathmandu resides in ward 10 [3]. Ninety-two persons were not able to be contacted because of lack of availability and 43 persons refused to provide information. Of the remaining 1,404 older adults, 332 were not living with at least one married son, 86 refused participation in the study, 101 admitted that they could not communicate well in Nepali, 58 terminated the interview in the middle, and 338 had more than one older adult in the household. The final sample size was 489 (247 men & 242 women).

### Ethical considerations

The Institutional Review Board of the Graduate School of Medicine, University of Tokyo approved the research study. Verbal consent was obtained because of the high rate of illiteracy and inability of subjects to sign their name. Previous studies have also utilized verbal informed consent [36]. Participants were informed about the study and that they could discontinue the interview at any time.

### Measurement

#### Dependent variables

The Geriatric Depression Scale (GDS), that was developed specifically for older adults, was used to measure depression in the study [37]. The major strength of the scale is its simplicity of administration and scoring. It uses a "yes-no" response format, with each indicator of depression each counting 1 point for a possible score of 0 to 30 points. A higher score means a higher level of depression. The GDS has a well-established internal consistency, with Cronbach's alpha reliabilities of 0.94 [37],0.91 [38], 0.87 [39], and 0.92 [40]. A Cronbach reliability coefficient of 0.93 was obtained in the present study. The GDS has been widely used in a number of health and service settings [41]. In a study among cognitively unimpaired geriatric clinical outpatients in Nepal, 83.3% older adults screened depressed using the GDS were also diagnosed depressed using the International Classification of Diseases (ICD)-10 Diagnostic Criteria for Research (DCR), illustrating a good sensitivity of the GDS [42]. The GDS has been used and translated into 24 different languages [43].

The second dependent variable used in the study was the Satisfaction With Life Scale (SWLS) [44]. It is a five-item scale, designed to measure the overall self-judgments of the respondents' satisfaction with life. The original seven-point Likert scale was reduced to five-points ranging from very dissatisfied (1) to very satisfied (5) so that it is easier for face-to-face interviews with mainly illiterate older Nepalese adults. The possible range of scores was from 5 (lowest SWLS) to 25 (highest SWLS). The scale has been tested for reliability and validity internationally [45]. The internal consistency, Cronbach alpha reliability in previous studies ranged from 0.79 to 0.89 [45,46]. The Cronbach's reliability coefficient for the SWLS in this study was 0.83. Test-retest reliability was 0.426 during one week interval (n = 10). The result of a principal component analysis was consistent with a single factor, with the first component explaining 64.6% of the total variance. A structural equation modelling also confirmed a good fit of the one-factor model (chi-square = 18.0, DF = 5.0, GFI = 0.98, AGFI = 0.95, RMSEA = 0.07).

#### Independent variables

##### Leisure and religious activity

The leisure social activity domain was represented by three variables: talking with neighbours, visiting friends, and socializing with other people. These items have been used in previous studies [47]. The solitary leisure activity domain was measured by three variables: watching television and listening to the radio, playing cards, and participating in physical exercise. The religious activity domain was operationalized by two measures: saying prayers, and involvement in religious activities. Activity participation was measured by asking the older adults how often they were engaged at the moment in these activities: never = 1, occasionally (once/twice a week) = 2 and daily = 3.

Those variables commonly reported in the literature that has impact on mental health (depression and satisfaction with life) of older adults were selected as control variables in the present study. The sociodemographic characteristics include age (continuous), gender (male = 1); marital status (married = 1); education (illiterate = 1, literate but no formal schooling = 2, schooling through high school = 3, college & above = 4).

Financial satisfaction was measured with a question, "compared to other people of your age in your neighbourhoods, are you financially satisfied?" Response options ranged from very dissatisfied to very satisfied. They were categorized very dissatisfied & satisfied = 1, fairly satisfied = 2, and satisfied & very satisfied = 3. Respondents were asked if they currently possessed inherited property or not (yes = 1).

##### Health variables

Self reported general health (SRH) was measured on a five-point Likert scale. SRH was elicited by asking "In general, how do you describe your health? [48]." The responses were categorized as, very bad and bad = 1, fair health = 2, and good and very good = 3. The SRH has been considered a predictor of mortality above and beyond "objective" measures of health status, behaviours, and socioeconomic status [49]. Measuring health by self assessed general health is reported to be a valid measurement for other countries as well [50].

##### Social support

Social support was defined as support from the son and provided to the son during the past year. Social support was measured by emotional support (listen when need to talk, share most private worries, and receive good advice at times of crisis) and instrumental support (financial assistance, home repair and household work, and transportation). The response options were "yes = 1" for support received/provided and "no = 0," for support not received/provided. The Cronbach reliability coefficient was 0.80 in this study.

### Translation

All the scales were translated into Nepali from English and back translated into English by professional translators. The scales and data collection instruments were pilot tested on a Nepali sample of 50 community-dwelling older adults for cultural sensitivity and reliability. The instrument was revised based on results of the pilot study.

### Statistical analysis

Descriptive statistics and the gender distribution of the sample was analyzed using t-test, chi-square test, and the Mann-Whitney's U Test (Table 1). ANOVA was used to examine the gender differences in activity participation and mental health (Tables 2 and 3). As for some variables had a small group size in Tables 2 and 3, literature points the need to be aware of the possibility of a non-significant result due to insufficient power [51]. The present study has relatively large sample size, and the group sample does not violate the SPSS calculations for ANOVA. Age, marital status, financial satisfaction, education, perceived health, support received and provided were used as confounding variables and results were analyzed using multiple regression. Separate multiple regressions were conducted for men and women. In the multiple regression model, the activity variables were entered simultaneously with the confounding factors (Tables 4 and 5). Statistical Package for Social Sciences (SPSS) version 15.0 (Chicago, IL, USA, 2006) for Windows was used for statistical analysis.

**Table 1:** Characteristics of the sample

	Total (n = 489)	Men (n = 247)	Women (n = 242)	*p*
	n (%)	n (%)	n (%)	
*Age in years*^*a *^*(mean ± SD)*	69.9 ± 8.1	70.5 ± 8.1	69.4 ± 8.3	0.153
*Marital status*^*b *^*(married)*	264 (54.0)	177 (71.9)	87 (36.0)	**0.000**
*Financial satisfaction*^*c*^				**0.032**
Dissatisfied	84 (17.2)	35 (14.2)	49 (20.2)	
Fair	241 (49.3)	120 (48.6)	121(50.0)	
Satisfied	164 (33.5)	92 (37.2)	72 (29.8)	
*Possess inherited property*^*b *^*(yes)*	396 (81.0)	216 (87.4)	180 (74.4)	**0.000**
*Education*^*c*^				**0.000**
Illiterate	277 (56.6)	79 (32.0)	198 (81.8)	
No schooling but literate	67 (13.7)	54 (21.9)	13 (5.4)	
High school	109 (22.3)	82 (33.2)	27 (11.2)	
College & above	36 (7.4)	32 (13.0)	4 (1.7)	
*Perceived health*^*c*^				0.198
Poor	248 (50.7)	120 (48.6)	128 (52.9)	
Fair	196 (40.1)	99 (40.1)	97 (40.1)	
Good	45 (9.2)	28 (11.3)	17 (7.0)	
*Social support received*^*a *^*(mean ± SD)*	4.1 ± 1.9	4.0 ± 1.9	4.2 ± 1.9	0.427
*Social support provided*^*a *^*(mean ± SD)*	3.0 ± 1.7	3.2 ± 1.7	2.8 ± 1.6	**0.023**
*Mental health*				
GDS^a^(mean ± SD)	11.3 ± 8.0	9.7 ± 7.5	12.9 ± 8.2	**0.000**
SWLS^a^(mean ± SD)	15.4 ± 3.6	15.6 ± 3.8	15.1 ± 3.5	0.121

SD: Standard Deviation ; : t-test ; : Chi-square test ; : Mann-Whitney's U Test

GDS : Geriatric Depression Scale (score ranges from 0 to 30)

SWLS : Satisfaction With Life Scale (score ranges from 5 to 25)

Score for social rupport received and provided ranges from 0 to 6.

Bold denote significant level at p < 0.05

**Table 2:** Bivariate analysis on activities and GDS

	Men (n = 247)			Women (n = 242)		
Activity variables	n (%)	GDS scale	*p *^*1*^	n (%)	GDS scale	*p *^*1*^

*Talk with neighbours*			**0.001**			0.280
Never	13 (5.3)	14.38		18 (7.4)	14.56	
Sometimes	121 (49.0)	10.79		154 (63.6)	13.19	
Daily	113 (45.7)	7.96		70 (28.9)	11.81	
*Visit friends*			**0.000**			**0.002**
Never	11 (4.5)	19.82		29 (12.0)	17.76	
Sometimes	182 (73.7)	9.53		191 (78.9)	12.28	
Daily	54 (21.9)	8.15		22 (9.1)	11.82	
*Socialize with others*			**0.000**			**0.000**
Never	31 (12.6)	17.48		47 (19.4)	17.57	
Sometimes	196 (79.4)	8.58		188 (77.7)	11.87	
Daily	20 (8.1)	8.45		7 (2.9)	9.00	
*Watch TV, Radio*			**0.000**			**0.000**
Never	7 (2.8)	15.57		10 (4.1)	24.20	
Sometimes	67 (27.1)	13.75		89 (36.8)	15.46	
Daily	173 (70.0)	7.87		143 (59.1)	10.50	
*Play cards*			0.243			0.519
Never	163 (66.0)	10.21		229 (94.6)	13.03	
Sometimes	77 (31.2)	8.84		11 (4.5)	10.64	
Daily	7 (2.8)	6.71		2 (0.8)	9.00	
*Do physical exercise*			**0.000**			**0.000**
Never	124 (50.2)	12.74		166 (68.6)	14.55	
Sometimes	65 (26.3)	7.08		53 (21.9)	9.96	
Daily	58 (23.5)	6.07		23 (9.5)	7.70	
*Say prayers*			**0.000**			**0.002**
Never	5 (2.0)	18.00		5 (2.1)	23.20	
Sometimes	49 (19.8)	12.76		37 (15.3)	15.08	
Daily	193 (78.1)	8.69		200 (82.6)	12.23	
*Religious activities*			**0.000**			**0.005**
Never	7 (2.8)	20.57		14 (5.8)	18.71	
Sometimes	156 (63.2)	9.33		144 (59.5)	13.17	
Daily	84 (34.0)	9.43		84 (34.7)	11.44	

GDS: Geriatric Depression Scale,(scores range from 0–30) ; *p *^1^: F-test (ANOVA)

Bold denote significant level at p < 0.05

**Table 3:** Bivariate analysis on activities and SWL

	Men (n = 247)			Women (n = 242)		
Activity variables	n (%)	SWLS	*p *^*1*^	n (%)	SWLS	*p *^*1*^

*Talk with neighbours*			**0.004**			0.240
Never	13 (5.3)	13.46		18 (7.4)	14.39	
Sometimes	121 (49.0)	15.12		154 (63.6)	14.94	
Daily	113 (45.7)	16.39		70 (28.9)	15.66	
*Visit friends*			**0.000**			**0.000**
Never	11 (4.5)	12.45		29 (12.0)	13.07	
Sometimes	182 (73.7)	15.24		191 (78.9)	15.22	
Daily	54 (21.9)	17.52		22 (9.1)	16.77	
*Socialize with others*			**0.000**			**.000**
Never	31 (12.6)	15.14		47 (19.4)	11.80	
Sometimes	196 (79.4)	14.06		188 (77.7)	13.93	
Daily	20 (8.1)	16.24		7 (2.9)	16.06	
*Watch TV, Radio*			**0.000**			**0.000**
Never	7 (2.8)	15.14		10 (4.1)	11.80	
Sometimes	67 (27.1)	14.06		89 (36.8)	13.93	
Daily	173 (70.0)	16.24		143 (59.1)	16.06	
*Play cards*			0.078			0.077
Never	163 (66.0)	15.33		229 (94.6)	15.03	
Sometimes	77 (31.2)	15.97		11 (4.5)	15.64	
Daily	7 (2.8)	18.29		2 (0.8)	20.50	
*Do physical exercise*			**0.000**			**0.001**
Never	124 (50.2)	14.73		166 (68.6)	14.70	
Sometimes	65 (26.3)	15.86		53 (21.9)	15.30	
Daily	58 (23.5)	17.24		23 (9.5)	17.52	
*Say prayers*			0.052			**0.044**
Never	5 (2.0)	14.00		5 (2.1)	12.20	
Sometimes	49 (19.8)	14.57		37 (15.3)	14.30	
Daily	193 (78.1)	15.92		200 (82.6)	15.33	
*Religious activities*			**0.009**			**0.002**
Never	7 (2.8)	12.71		14 (5.8)	12.71	
Sometimes	156 (63.2)	15.29		144 (59.5)	14.85	
Daily	84 (34.0)	16.45		84 (34.7)	15.93	

SWLS: Satisfaction With Life Scale,(scores range from 5–25); *p *^*1 *^: F-test (ANOVA)

Bold denote significant level at p < 0.05

**Table 4:** Multivariate analysis on Geriatric Depression Scale

	Men (n = 247)		Women (n = 242)	
	B	*p*	B	*p*

Social activities (1–3)				
Talk with neighbors	-0.61	0.412	0.88	0.289
Visit friends	0.98	0.312	-1.20	0.270
Socialize with others	-1.23	0.236	-1.45	0.195
Watch television & listen radio	-1.88	**0.015**	-2.68	**0.001**
Play cards	0.44	0.564	-0.29	0.854
Do physical exercise	-1.05	**0.048**	-0.89	0.235
Religious engagement (1–3)				
Say prayers	-2.75	**0.001**	-0.50	0.644
Religious activities	0.56	0.459	0.45	0.601

R Square	0.48		0.43	
R Square Change	0.07	**0.000**	0.06	**0.004**
Adjusted R Square	0.45		0.40	

Multiple regression analysis was conducted ; B : Unstandardized B co-efficient

Bold denote significant level at *p *< 0.05

(Control variables include age, marital status, financial satisfaction, education, perceived health, social support received and provided)

**Table 5:** Multivariate analysis on Satisfaction With Life Scale

	Men (n = 247)		Women (n = 242)	
	B	*p*	B	*p*

Social activities (1–3)				
Talk with neighbours	-0.13	0.758	-0.30	0.422
Visit friends	1.02	0.063	1.29	**0.009**
Socialize with others	1.22	**0.038**	1.45	**0.005**
Watch television & listen radio	0.19	0.666	0.92	**0.016**
Play cards	-0.12	0.780	0.90	0.215
Do physical exercise	-0.17	0.564	-0.18	0.594
Religious engagement (1–3)				
Say prayers	0.17	0.718	-0.08	0.877
Religious activities	0.53	0.217	-0.12	0.755

R Square	0.35		0.36	
R Square Change	0.06	**0.013**	0.10	**0.000**
Adjusted R Square	0.31		0.32	

Multiple regression analysis was conducted ; B : Unstandardized B co-efficient

Bold denote significant level at *p *< 0.05

(Control variables include age, marital status, financial satisfaction, education, perceived health, social support received and provided)

## Results

### Description of the sample

Table 1 shows the socio-demographic characteristics of the study sample. The sample was 247 (50.5%) men and 242 (49.5%) women. In this study, divorced/separated and single were very few in number, and were included with widow/er. A significant gender difference was found for marital status (chi square test, value = 61.3; p < 0.0001) with a higher number of widowed women. Fourteen percent men were dissatisfied with their financial status compared to 20.2% of the women (p < 0.05). Men (87.4%) significantly possessed inherited property compared to women (74.4%) (p < 0.0001). Illiteracy was higher among women (81.8%) compared to men (32.0%), (p < 0.0001). Men (3.2 ± 1.7) significantly provided more support to son than women (2.8 ± 1.6) (p < 0.05). Women reported significantly higher scores on the GDS (p < 0.0001).

### Activity participation and depression and satisfaction with life

An ANOVA demonstrated that those older adults who did not participate in activities scored significantly higher on the GDS scores and have lower scores on the satisfaction with life scale (Tables 2 and 3). The majority of older adults in the sample reported that they spoke to their neighbours occasionally (men = 49.0%; women 63.6%), visited friends on an occasional basis (men = 73.7%; women = 78.9%), socialized occasionally (men = 79.4%; women = 77.7%), and were occasionally involved in religious activities (men = 63.2%; women = 59.5%). The majority of the men in the sample reported saying prayers daily (men = 78.1%; women = 82.6%), listened to the radio and watched television daily (men = 70.0%; women = 59.1%). Similarly respondents never played cards (men = 66.0%; women = 94.6%) and never participated in any physical activities (men = 50.2%; women = 68.6%). An ANOVA demonstrated that there was a significant difference in activity involvement between men and women, with greater men involvement in talking with neighbours (p = 0.001), visiting friends (p = 0.000), socializing with others (p = 0.000), playing cards (p = 0.000), and participating in physical activities (p = 0.000). There were no significant gender differences in saying prayers and participation in religious activities.

### Activity participation and depression

Standard multiple regression (Table 4) was used to explore if involvement in leisure and religious activities helped relate to depression in older adults. Separate regression models were developed to explore the gender differences in correlates of depression. Watching television and listening to the radio (B = -1.88; p < 0.005), participating in physical exercise (B = -1.05; p < 0.05) and saying prayers (B = -2.75; p < 0.005) were significant correlates of depression among men. Saying prayers was the strongest activity correlating to depression in men with those men who prayed regularly likely to score lower on the depression scale. In the female regression model, watching television and listening to the radio (B = -2.68; p < 0.005) was the only significant activity participation correlate. The R square change was significant for both men (p < 0.0001) and women (p < 0.005) regression models.

### Activity participation and Satisfaction with life (SWLS)

Standard multiple regression (Table 5) was used to explore whether involvement in social and religious activities correlated to satisfaction with life in older adults in Nepal. Separate regression models for gender identified socializing with others (B = 1.22; p < 0.05) to be a significant correlate for men, while visiting friends (B = 1.29; p < 0.05), socializing with others (B = 1.45; p < 0.005), and watching television and listening to the radio (B = .92; p < 0.05) emerged as significant correlates for women.

## Discussion

Activities in late-life have been found to have positive effects on the mental health of the older adults. The goal of this study was to explore whether leisure and religious activity participation correlated to depression and satisfaction with life in older adults in a developing country. The confounding factors of age, marital status, financial satisfaction, education, perceived health, social support from the son and provided to the son were controlled. Saying prayers, watching television and listening to the radio, and participating in physical exercise correlated to lower levels of depression for men. For women, only watching television and listening to the radio correlated to lower the levels of depression. Saying prayers was not a significant correlate of depression for women. In Nepal, where the Hindu religion is prevalent, men are expected socially to be priests. The regression analyses demonstrated that the influence of religious and activity participation on depression in older Nepalese adults is gender specific.

Wide spread use of television and FM radio stations in Kathmandu occurred after 1990 [52]. Television is a primary means of entertainment with 64.6% of the older adults watching television and listening to the radio daily in this study. There are few other means of entertainment in Nepal. Religious programming on television has also become more popular in Nepal [53]. Watching television and listening to the radio are also the most popular leisure activities among the older adults in other countries. A study in Japan found that the pleasure of watching television correlated to higher happiness scores for older adults in that country [54]. An eight year longitudinal study of older adults in Canada found that watching television was the key activity that older adults participated in later life [55].

Women, who were significantly more depressed than men may not place participating in outdoor activities as a high priority. Previous literature introduces the possibility of reciprocal causation in that depression may cause lower levels of social engagement. For example, a study revealed that depression in older adults may be due to loss of interest in previously valued social roles [56]. Longitudinal studies have shown that social engagement protects against depression [57]. Depression and social participation varies from one individual to another. Further longitudinal studies about activity participation and mental health in developing Asian countries are necessary to establish causal relationships.

One of the important leisure activities out of the home for older adults who have social networks is being with relatives and friends [58]. Conversation with family or neighbours has been shown to have a significant association with happiness in older adults [54]. Activities like frequency of going outdoors were found useful indicators that predict functional and psychological changes among the ambulatory frail older adults living at home in Japan [59]. This study is consistent with the findings that socializing with others is a significant correlate of higher satisfaction with life. A study among Spanish older adults showed that engagement with friends seemed to be protective for cognitive decline in women, but not in men [60]. In the present study also visiting friends predicted satisfaction with life for women only.

Though playing cards was not a significant correlate of depression or satisfaction with life, 88 (18.0%) respondents occasionally played cards. Playing cards in Nepal is an informal means of recreation where people from the same community gather at someone's house. Notably men played cards more frequently than women. Among those who played cards occasionally, 87.5% were men and 12.5% were women. Gambling or casino activity has been a socially acceptable activity in some societies for older adults and it is seen as a social rather than a risky behaviour [61]. One of the reasons for lower participation in Nepal might be due to the fact that playing cards as a mean of gambling is a socially unaccepted behaviour.

Further research is necessary to explore card playing among older adults in Nepal. The social and religious dogma in Nepali society is that as people age, they should refrain from many social activities. This may have affected the reporting of the social activities and result in under-reporting. Though household activities were not identified in the study, it is possible that the older adults, particularly women, were more engaged in household activities and less engaged in out of home activities.

The variables that correlated to depression in this study were not significant correlates of satisfaction with life in the total sample. Leisure social activity variables, such as visiting friends and socializing with others, were important correlates of life satisfaction. Perceived health and perceived financial satisfaction were significant factors affecting the mental health of the older adults. Poverty has a strong positive relationship in poorer older adults in developing countries (as in other countries too) are less privileged as the existing health and pension programs are not relevant to them [62].

Men were found to participate in physical exercise more frequently than women. Literature supports that older adults who are engaged in regular physical activities have lower prevalence of depression [18]. The results of this study support that participating in physical exercise is a significant predictor of lower levels of depression (Table 4). This might be the reason why participating in physical exercise was significant correlate for men and not for women in lowering the depression. Social contact is associated with physical activity in improving the well-being of the older adults [63].

Participation in cultural events is also an important activity for older adults. This activity was not directly investigated in the study, however, in Nepali Hindu society, cultural events are considered to be religious activities. Questions regarding involvement in religious activities and saying prayers partially addressed cultural events in Nepal. There was no significant gender difference in religious participation. Because of the Hindu cultural norms that men perform the religious rites, women did not express themselves as performing religious activities, though women were also equally involved in these activities.

The gender analysis was considered an important aspect of this study. Nepalese society is marked by a distinct separation of social roles by gender. There are social and cultural norms about social behaviour and networks that are distinctly different for men and women. Older women's social role in Nepal is restricted to household life, whereas the male role is more broadly centred on social networks. Literature supports gender-specific associations between social relationships and health for men and women [21]. Data from this study suggests that social relations outside the family were not frequent for older women in Nepal and that solitary activities such as watching television and listening to the radio were associated with lower levels of depression.

The cross-sectional nature of research is a major limitation of the study as it cannot confirm the causal relationships between study variables. Longitudinal studies are warranted in future studies. Although the principal component analysis and structural equation modelling illustrated a good construct validity and reliability coefficient was sufficiently high for the SWLS, because the response options were reduced to five-point Likert scale it reduces the variability in scores. As the study focuses on the older adults living in an urban area of Nepal, it might not necessarily be generalizable to other areas. The present study focused on informal and religious activities, but research on formal activities and volunteering activities needs to be explored in future research.

## Conclusion

Leisure social activities were only found to increase the satisfaction with life in older adults, but were not correlates of lower levels of depression. The solitary activity of watching television and listening to radio was found to be a significant correlate of depression. The reasons why women were more engaged in solitary activities than men needs to be explored further in future research.

Engagement in activities has shown positive effects in the mental health of older adults. The findings can be useful for planners and policy makers that emphasizes on informal and solitary activities can help reduce the level of depression and thus better quality of life of older adults. Medical costs are substantially lowered for older adults who are active [64].

## Competing interests

The author(s) declare that they have no competing interests.

## Authors' contributions

RG was responsible for the conception, design, and analysis of the study. All authors participated in interpretation of the findings. RG drafted the manuscript. TS and IK revised and commented on the draft and all authors read and approved the final version of the paper. All authors confirm that the content has not been published elsewhere and does not overlap or duplicate their published work.

## Pre-publication history

The pre-publication history for this paper can be accessed here:


